# Effects of oral care combined with neuromuscular electrical stimulation on clinical outcomes in the acute phase of acute ischemic stroke: a pilot randomized controlled trial

**DOI:** 10.1186/s12984-025-01652-6

**Published:** 2025-05-30

**Authors:** Yi-Ting Huang, Chia-Chun Tang, Chen-Chih Chung, Chi-Hsiang Chung

**Affiliations:** 1 Department of Neurology, Shuang Ho Hospital, Taipei Medical University, New Taipei City, Taiwan; 2 School of Nursing, College of Medicine, National Taiwan University, 10051 No. 1, Sec. 1, Jen-Ai Road, Taipei, Taiwan; 3 Department of Nursing, National Taiwan University Hospital, Taipei, Taiwan; 4 Taipei Neuroscience Institute, Taipei Medical University, Taipei, Taiwan; 5 Department of Neurology, School of Medicine, College of Medicine, Taipei Medical University, Taipei, Taiwan; 6 School of Public Health, National Defense Medical Center, Taipei, Taiwan; 7 Data Analysis and Management Center, Department of Medical Research, Tri-Service General Hospital, Taipei, Taiwan

**Keywords:** Acute cerebral infarction, Dysphagia, Stroke-associated pneumonia, Transcutaneous neuromuscular electrical stimulation, Oral care, Rehabilitation

## Abstract

**Background:**

Stroke-associated dysphagia significantly increases the risk of pneumonia in persons with acute ischemic stroke (AIS), yet effective early interventions remain limited. This pilot randomized controlled trial examined the feasibility and clinical effects of a nurse-delivered combined intervention involving neuromuscular electrical stimulation (NMES) and comprehensive oral care—including toothbrushing using the Bass method, tongue cleaning, and moisturizing twice daily—during the acute stroke phase.

**Methods:**

This randomized, parallel group pilot trial assigned persons with AIS into three groups: (i) oral care only, (ii) oral care + NMES, and (iii) standard care (control). Interventions began within 48 h of stroke onset and continued twice daily for five days, starting within 48 h of stroke onset. Outcome measures, including the Revised Oral Assessment Guide (ROAG) and Gugging Swallowing Screening (GUSS), were assessed at baseline, day 4, and day 8 post-stroke. Statistical analysis employed one-way analysis of variance (ANOVA), chi-square tests, and generalized estimating equations (GEE) to analyze group differences and longitudinal trends.

**Results:**

Among 35 participants (mean age 68.3 ± 12.5 years; 51.4% men), both intervention groups demonstrated significant improvements in swallowing and oral health outcomes over time, compared to standard care (*p* < 0.05). Although this pilot study was not powered to determine superiority between the two intervention groups, the oral care + NMES group demonstrated the greatest improvements in GUSS and ROAG scores.

**Conclusion:**

Findings from this pilot trial support the feasibility of nursing staff implementing combined oral care and NMES within 48 h of AIS onset. The results highlight the potential for meaningful clinical benefits, particularly in settings with limited access to specialized rehabilitation. Larger, blinded, multi-center trials are warranted to further evaluate the efficacy and broader applicability of this early intervention strategy.

**Registration:**

The study protocol was registered in the Protocol Registration and Results System (PRS) under ID N202108021 as a supplementary registration due to initial unfamiliarity with PRS registration requirements, with the registration date recorded as 11/14/2024.

## Introduction

Stroke-associated pneumonia (SAP) affects approximately 10% of hospitalized persons with stroke [1, 2]. It is the leading cause of mortality among post-stroke complications, with 94% of cases occurring within the first seven days of admission [3]. SAP prolongs hospital stays, delays functional recovery, and reduces post-stroke quality of life.

Several post-stroke conditions may contribute to the development and severity of SAP, including dysphagia, cognitive impairment, and poor oral hygiene. Dysphagia—a primary risk factor for SAP—significantly increases aspiration risk due to impaired swallowing and reduced clearance of respiratory secretions [4]. Cognitive impairment or reduced consciousness can delay pneumonia recognition and increase the risk of respiratory infections [5]. Up to 65% of stroke survivors experience dysphagia, with a threefold greater aspiration risk [6]. Those with aspiration have an elevenfold higher risk of developing pneumonia.

Poor oral hygiene contributes to SAP risk by promoting bacterial overgrowth in the oral cavity, which may be aspirated into the lungs [7]. Altered in the oral microbiota—due to poor hygiene or impaired salivary clearance—not only increases the risk of aspiration-related infections but may also worsen dysphagia through oral inflammation, discomfort, and impaired neuromuscular coordination [5, 8]. These findings underscore the importance of comprehensive oral care in preventing respiratory infections and supporting dysphagia management during stroke recovery.

Despite the clinical burden of SAP, consensus on prevention strategies remains limited. Prophylactic antibiotics have shown variable outcomes in reducing SAP incidence and improving long-term recovery, with some studies indicating no significant impact on mortality rates [9–11]. Neuromuscular electrical stimulation (NMES) has emerged as a potentially beneficial intervention for post-stroke dysphagia by enhancing neuromuscular coordination and restoring swallowing ability. NMES, applied to throat and masticatory muscles, has been associated with improved oral intake, reduced symptom severity, and enhanced quality of life in persons with stroke. Furthermore, systematic reviews suggest that NMES may offer superior outcomes compared to conventional non-stimulation-based interventions in improving swallowing function [4, 12, 13].

Although previous studies have reported promising results, key gaps remain—particularly regarding the timing of NMES and its role in preventing SAP. Most research has focused on the subacute or chronic phases of stroke recovery, with limited investigation of NMES during the acute phase (within 48 h of stroke onset). Yet, both SAP risk and neuroplastic potential are highest in the first week post-stroke. Warusevitane et al. (2015) found that 94% of post-stroke pneumonia cases occur within the first week of hospitalization, underscoring the importance of targeting this critical window to reduce complications and improve recovery outcomes [14–17]. Additionally, the potential benefit of combining NMES with comprehensive oral care in the acute phase has not been previously explored. Since oral care reduces oral bacterial load—a modifiable SAP risk factor—investigating their combined use may offer additional advantages for early dysphagia management. Second, while NMES has shown improvements in swallowing function, few studies directly assess its impact on SAP incidence. Given SAP’s significant morbidity and mortality, evaluating this outcome is essential. To address these gaps, our study implemented a combined intervention of NMES and comprehensive oral care during the acute stroke phase. This approach was designed not only to improve swallowing function but also to potentially reduce SAP risk. While definitive effects on SAP incidence require validation in more extensive trials, the pilot results support its feasibility and clinical promise.

Access to speech-language pathologists is often limited during the early hospitalization phase, especially in acute stroke units. Therefore, we aimed to examine whether a nurse-delivered, low-threshold intervention could be initiated early to promote swallowing recovery and reduce preventable complications such as SAP. Given these limitations, recent studies suggest that NMES may exert neuromodulatory effects even in the absence of active swallowing, providing a rationale for its application during passive interventions such as nurse-led oral care. Although passive in nature, the combined use of oral care and NMES was selected for its practicality, safety, and theoretical potential to stimulate both sensory and motor pathways—even in this population not yet able to engage in active swallowing exercises.

## Study design and methods

This study was a randomized controlled trial (RCT) with a parallel group design.

### Setting and participants

The trial was conducted at a medical center in Northern Taiwan between November 2021 and May 2023. Eligible participants met the following inclusion criteria:

1. Aged ≥ 20 years.
2. First-ever diagnosis of acute ischemic stroke (AIS).
3. Stroke onset within 48 h of enrollment.
4. National Institutes of Health Stroke Scale (NIHSS) score ≥ 5 upon admission, with at least one of the following items affected: facial palsy, language, or dysarthria.
5. Swallowing difficulty, indicated by a Gugging Swallowing Screen (GUSS) Indirect Test score < 5.
6. Ability to communicate verbally or in writing and provide informed consent.

Exclusion criteria were as follows:

1. Presence of pneumonia or clinical symptoms of infection upon admission.
2. Requirement for mechanical ventilation.
3. Antibiotic or immunosuppressant use within the past month.
4. Eligibility for intravenous recombinant tissue plasminogen activator (r-tPA) or mechanical thrombectomy, as these individuals required intensive care unit (ICU) admission where structured oral hygiene was routinely administered by nursing staff. This pre-existing care could confound the intervention effect and compromise consistency.
5. History of seizure or epilepsy.
6. Oral-pharyngeal tumors or a history of extensive surgery or radiotherapy to the head and neck.
7. Use of electrically sensitive biomedical devices (e.g., pacemakers or defibrillators).
8. Pregnancy.

As a pilot study aimed at assessing feasibility, the target sample size was set at 30 based on a similar prior study [18]. Participants were randomly assigned to groups using a computer-generated randomization sequence.

### Data collection

Eligible participants were screened and recruited daily based on inclusion and exclusion criteria. Consenting participants were randomly allocated to three groups: **Group A (Oral Care Group)**,** Group B (Oral** C**are + NMES Group**,** O-NMES)**,** and Group C (Control Group). The randomization list was generated via SPSS and then sealed in an envelope to ensure allocation concealment. Due to limited human resources in this pilot study**,** blinding was not feasible.** Interventions were terminated if participants developed SAP, required ventilator support, died, or were discharged. Data were collected at three pre-defined time points aligned with stroke onset. The baseline assessment (T0) was conducted within 48 h of stroke onset, followed immediately by the intervention, which was delivered twice daily for five consecutive days. A midpoint evaluation (T1) was conducted on the fourth day post-stroke to capture early changes, and the final assessment (T2) was performed on day 8. All participants remained hospitalized throughout the study.

The timing of outcome assessments was strategically aligned with the high-risk period for SAP, defined as the first 7 days following stroke onset according to the 2014 Manchester consensus [3, 19]. SAP diagnosis was based on the Centers for Disease Control and Prevention (CDC) criteria and confirmed by attending physicians. It was defined as the presence of respiratory symptoms (e.g., dyspnea, rales), radiographic evidence of new infiltrates on chest X-ray, and systemic signs of infection, such as fever > 38 °C or purulent sputum, occurring during hospitalization [20]. All SAP diagnoses were documented in the medical records.

**Fig. 1:**
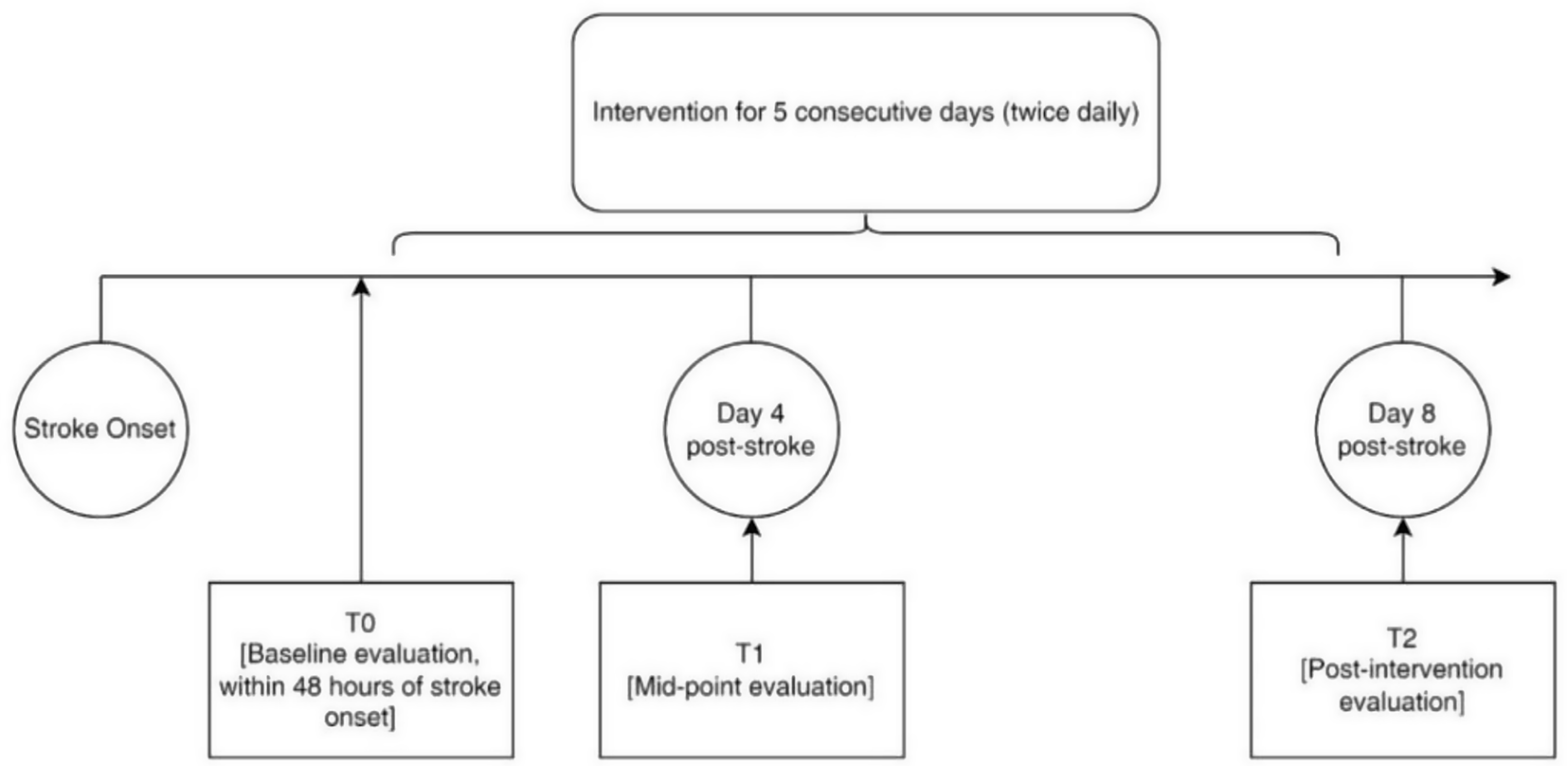
Study timeline and assessments points

Collected data included demographics, disease-related information, swallowing function improvements (primary outcome), and SAP incidence (secondary outcome). Demographic data (e.g., age, sex, cerebrovascular risk factors) and disease-related information (e.g., Glasgow Coma Scale [GCS], NIHSS, and biochemical data) were recorded only at T0 to describe the study sample and examine baseline comparability. Biochemical data included white blood cell (WBC) count, C-reactive protein (CRP), blood urea nitrogen (BUN), creatinine, albumin, and glucose levels, as these markers are commonly included in SAP risk models and reflect infection, inflammation, and nutritional status [22–24]. Nasogastric tube (NGT) insertion, including timing and aspiration events, was documented for participants as necessary during the study period. Primary outcomes were measured at all three time points, while secondary outcomes were collected at T2. A schematic timeline of the study phases is shown in Fig. 1 to illustrate the schedule of data collection time points and intervention duration.

### Intervention protocol

Participants in Group A (Oral Care) received structured oral care twice daily for five days. This care protocol, introduced by the first author, included four procedures: preparation, assessment, oral cleaning, and tongue cleaning. Preparation involved setting up tools and positioning the participant in an upright posture. Oral assessment and debris removal were performed using a flashlight and toothbrush. Toothbrushing followed the **Bass technique**, in which the toothbrush was angled at 45° toward the gingival sulcus and moved in gentle circular motions over two to three teeth at a time, followed by sweeping strokes along the tooth surface [25]. Each session used fluoridated toothpaste (> 1,000 ppm, < 0.5 cm³) and lasted approximately 2 min, consistent with plaque control recommendations for older adults [26]. After brushing, the anterior two-thirds of the tongue was cleaned using a moistened toothbrush.

**Fig. 2:**
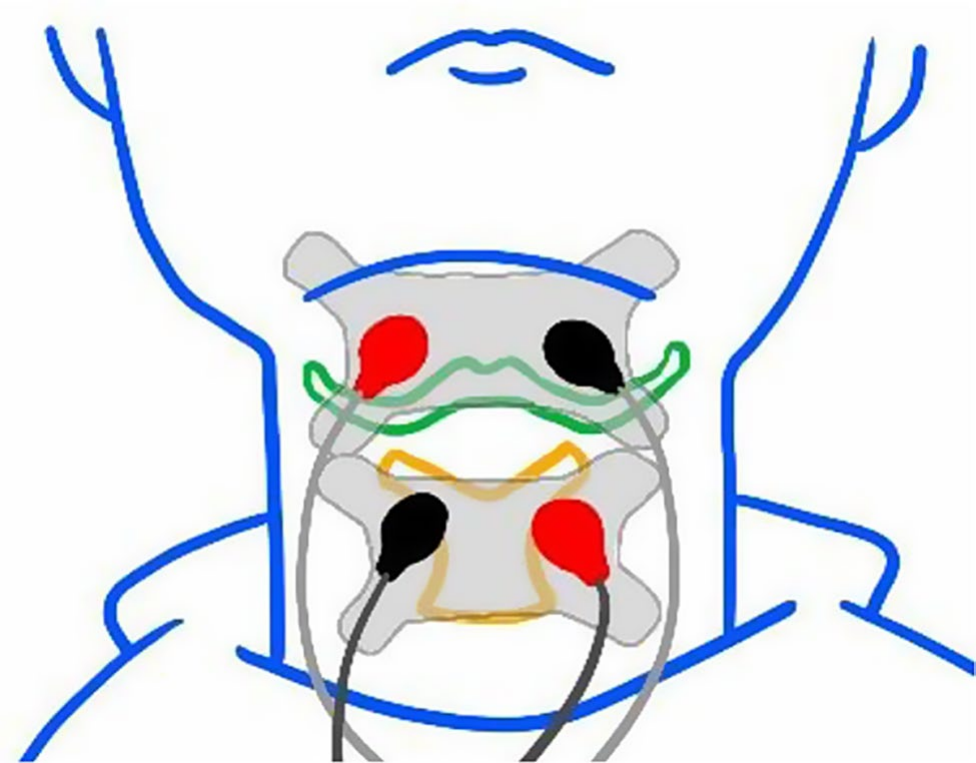
Electrode placement for NMES therapy

Participants in Group B (**O-NMES**) received the same oral care as Group A, with additional NMES therapy after each oral care session. Participants were instructed to shave facial hair and cleanse the neck skin with alcohol wipes before electrode placement. Four electrode pads were placed horizontally above and below the hyoid bone (Fig. 2) to provide dual-pole stimulation across four channels at a fixed pulse rate of 80 Hz and a fixed biphasic pulse duration of 300 µs. Each session lasted 30 min, with the stimulation intensity gradually increased in intervals of 0.5 mA, adjusted based on participants’ feedback, and adjusted to the maximum comfortable level for everyone. NMES was administered using VitalStim^®^ (Chattanooga, TN, USA).

Participants in Group C (Control) received standard care per the unit’s existing protocol, which consisted of daily verbal education provided to participants and their caregivers by nurses to encourage oral hygiene practices. However, no structured or supervised oral care was provided by staff.

### Instruments

Disease severity was assessed using the Glasgow Coma Scale (GCS) and the National Institutes of Health Stroke Scale (NIHSS). Swallowing function and oral hygiene were evaluated using the Revised Oral Assessment Guide (ROAG) and the Gugging Swallowing Screen (GUSS).

#### Glasgow Coma Scale (GCS)

A 3–15 point scale measuring eye, verbal, and motor responses, used to assess the level of consciousness at baseline. Higher scores indicate better neurological status. GCS was administered by trained nurses [27, 28].

#### National Institutes of Health Stroke Scale (NIHSS)

Comprising 15 neurological examination items widely used to assess stroke severity. Due to its simplicity, repeatability, reliability, and validity, NIHSS scores are a standard indicator in stroke models for evaluating AIS outcomes [29–31].

#### Revised Oral Assessment Guide (ROAG)

A validated instrument evaluating eight oral domains: voice, lips, mucosa, tongue, gums, teeth/dentures, saliva, and swallowing function. Each domain is scored from 1 (healthy) to 3 (severe problem), with total scores ranging from 8 to 24. Higher scores indicate poorer oral health [32, 33]. Prior studies have linked higher ROAG scores to increased aspiration risk. For example, Noguchi et al. reported that 68.4% of adults with mild to moderate oral problems and 94.5% with severe oral problems were at risk of aspiration. An AUC of 0.72 (95% CI: 0.60–0.84) was reported for ROAG as a predictor of pneumonia-related aspiration [34].

#### Gugging Swallowing Screen (GUSS)

A bedside dysphagia screening tool for acute persons with stroke, operates on a 0–20 point scale where higher scores indicate better swallowing function [35, 36]. The GUSS consists of two parts: the indirect swallowing screening (part 1) and the direct swallowing test (part 2). It provides dietary recommendations that range from typical diets to enteral feeding. GUSS has demonstrated excellent predictive validity, with an AUC of 0.93 (95% CI: 0.88–0.97). Its sensitivity is 0.97 (95% CI: 0.93–0.99), and specificity is 0.67 (95% CI: 0.59–0.74). Early use of GUSS by nurses has been shown to reduce pneumonia rates (*p* = 0.004) [37, 38]. GUSS was administered by trained nurses. In this study, the inclusion criteria were based on the first part of GUSS; however, the following assessments reflect the full 0–20 total from the complete GUSS assessment.

### Statistical analysis

All analyses followed an intention-to-treat (ITT) approach, including all randomized participants in the group to which they were originally assigned, regardless of protocol adherence. Categorical variables were frequencies (n) and percentages (%). Fisher’s exact test was used for comparisons due to the small sample size. Continuous variables were reported as means (M) and standard deviations (SD). Baseline characteristics and outcomes were compared across groups using one-way ANOVA or Kruskal Wallis tests for continuous variables and Chi-square or Fisher’s exact tests for categorical variables. Changes in NIHSS, ROAG, and GUSS scores over three time points (T0, T1, T2) were analyzed using Generalized Estimating Equations (GEE) to assess intervention effects. All analyses followed an intention-to-treat approach, including all randomized participants in their originally assigned groups, regardless of protocol adherence. Two-sided hypothesis testing was applied, with a significance threshold of *p* < 0.05.

## Results

### Demographics and baseline characteristics of the study cohort

The trial recruited participants from December 9, 2021, to March 24, 2023, with follow-up completed by March 30, 2023. During the data collection period, 37 hospitalized persons with AIS met the inclusion criteria. Two participants refused to participate in the trial due to their unwillingness to undergo intervention therapy. Ultimately, 35 participants (94.6%) were enrolled and randomly assigned, comprising 13 participants in Group A (experimental group receiving oral care), 10 in Group B (experimental group receiving oral care combined with NMES, O-NMES), and 12 in Group C (control group). Participants in Group C (Control) received standard care, consisting of daily verbal education on oral hygiene without structured or supervised oral care. Nursing logs indicated an average brushing frequency of 0.63 times per day, reflecting variability in routine practices.

**Fig. 3:**
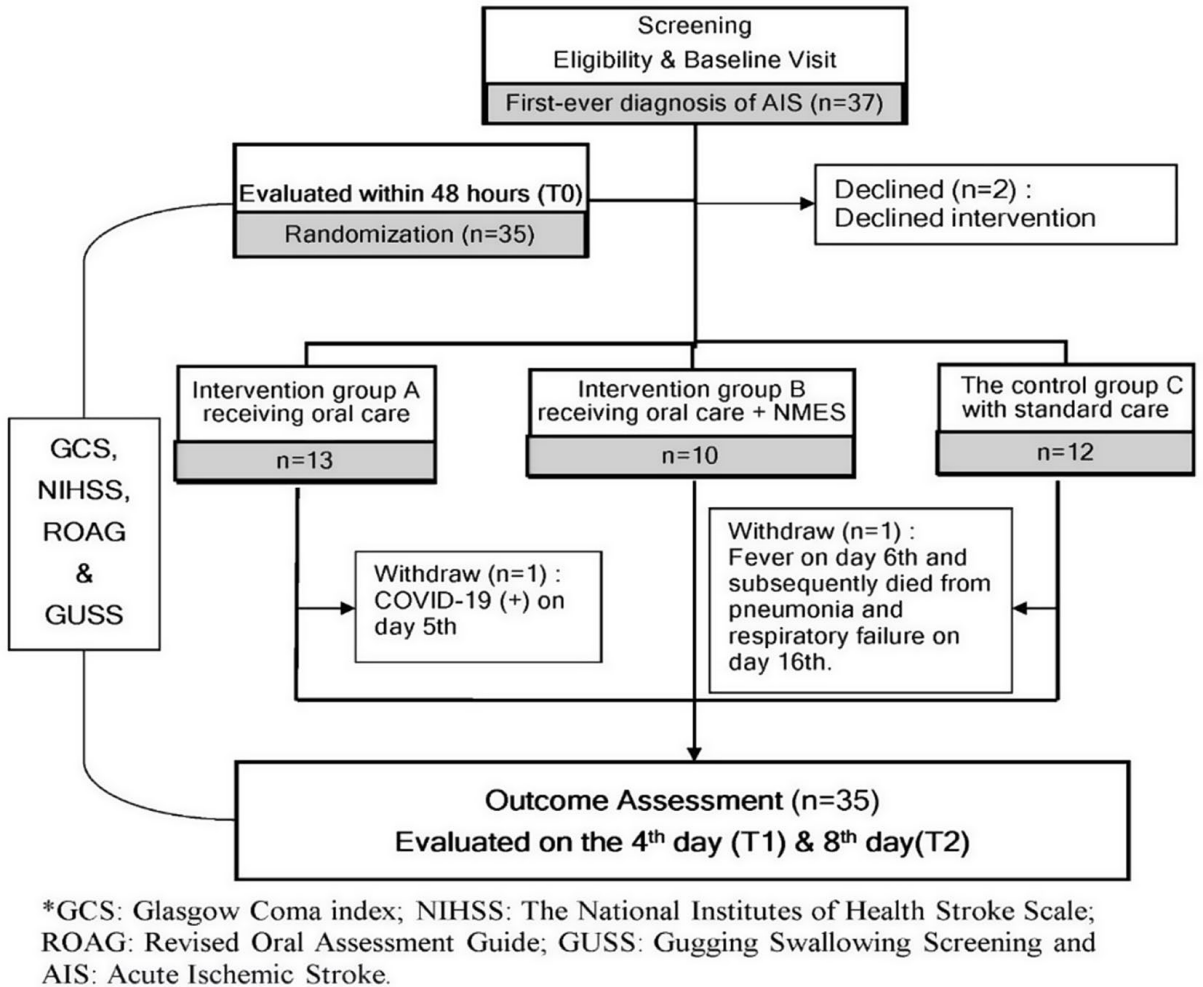
Flowchart of study design and participant allocation

Among the 35 randomized participants, two did not complete the full intervention period. One participant in the control group (Group C) developed a fever on day 6 and subsequently died from aspiration pneumonia and respiratory failure on day 16 of hospitalization. As this participant had completed all three scheduled assessments (T0, T1, and T2), their data was included in the intention-to-treat analysis. Another participant in Group B was transferred to coronavirus disease 2019 (COVID-19) quarantine on day 3 of stroke post-enrollment. Although the intervention was discontinued, the participant completed both T1 and T2 assessments. The participant remained asymptomatic aside from a positive COVID-19 rapid test, and chest X-ray findings did not indicate pneumonia. Their data were also included in the ITT analysis. These events have been clearly annotated in the participant flowchart (Fig. 3), and no adverse events related to the intervention protocol were observed during the study. ITT analysis included all 35 participants. In contrast, per-protocol (PP) analysis excluded the quarantined participant, resulting in 34 analyzed cases. No significant differences were found between ITT and PP analyses in baseline characteristics or pre-intervention outcomes. Subsequent results are presented based on ITT analysis.

The baseline characteristics of the study participants were categorized into two domains: demographic and disease-related variables (Table 1).

Among the 35 participants, ages ranged from 44 to 92 years (mean = 68.3 ± 12.5), with a majority being male (*n* = 18, 51.4%). Sixteen participants (45.7%) had a history of smoking. The most common vascular risk factors were hypertension (*n* = 25, 71.4%), hyperlipidemia (*n* = 15, 42.9%), and diabetes (*n* = 14, 40.0%), followed by atrial fibrillation (*n* = 6, 17.1%), coronary artery disease (CAD) (*n* = 5, 14.3%), heart failure (*n* = 3, 8.6%), and chronic obstructive pulmonary disease (COPD) (*n* = 2, 5.7%). Significant group differences were observed in age and the prevalence of CAD, with Group B being significantly older and Group C having more participants with CAD (*p* = 0.019 and *p* = 0.004, respectively); other demographic variables were not significantly different among groups.

The mean GCS score at baseline was 14.77 (SD = 0.97) across all participants, with no significant differences among groups (*p* = 0.175). Baseline NIHSS scores were also comparable among the three groups (*p* = 0.567). Seven (20.0%) participants had a NGT inserted at the baseline. Specifically, NGT insertion was observed in four participants (40.0%) in Group B (O-NMES), two (15.4%) in Group A (Oral Care), and one (8.3%) in Group C (Control). A chi-square test revealed no statistically significant difference in NGT distribution across the three groups (*p* = 0.158).

**Table 1:** Baseline characteristics of the participants

	Entire cohort (*n* = 35)	Intervention groups			*p*-value
		Oral care (Group A, *n* = 13)	Oral care + NMES (Group B, *n* = 10)	Control (Group C, *n* = 12)	
**Demographic information**					
**Age**	68.3 ± 12.5	66.5 ± 10.5	75.9 ± 6.1	64 ± 15.9	0.019*
**Female**	17 (48.6)	5 (38.5)	7 (70.0)	6 (41.7)	0.273
**BMI**	25.2 ± 3.8	24.9 ± 3.1	25.2 ± 4.3	25.5 ± 4.3	0.972
**Vascular risk**					
Hypertension	25 (71.4)	8 (61.5)	7 (70.0)	10 (83.3)	0.480
Dyslipidemia	15 (42.9)	5 (38.5)	6 (60.0)	4 (33.3)	0.417
Diabetes mellitus	14 (40.0)	5 (38.5)	4 (40.0)	7 (41.7)	0.987
Atrial fibrillation	6 (17.1)	1 (7.7)	3 (30.0)	2 (16.7)	0.371
Coronary artery disease	5 (14.3)	0 (0)	0 (0)	5 (41.7)	0.004**
Heart failure	3 (8.6)	1 (7.7)	1 (10.0)	1 (8.3)	0.980
COPD	2 (5.7)	1 (7.7)	0 (0)	1 (8.3)	0.653
Cigarette smoking	16 (45.7)	6 (46.2)	3 (30.0)	7 (58.3)	0.414
**Disease-related Information**					
**Biochemical Parameters**					
WBC (µL)	8564.0 ± 2383.6	8950.8 ± 2616.6	8134.0 ± 2610.5	8503.3 ± 2041.4	0.693
Neutrophils (%)	67.55 ± 12.16	67.68 ± 13.82	64.47 ± 11.64	69.98 ± 11.10	0.545
Creatinine (mg/dL)	0.935 ± 0.415	0.798 ± 0.082	1.125 ± 0.623	0.924 ± 0.391	0.243
Albumin (mg/dL)	3.93 ± 0.34	3.95 ± 0.29	3.80 ± 0.46	4.02 ± 0.26	0.385
Glycated hemoglobin (%)	6.51 ± 1.32	6.65 ± 1.53	6.06 ± 0.60	6.75 ± 1.53	0.700
**GCS**	14.77 ± 0.97	14.38 ± 1.56	15 ± 0	15 ± 0	0.175
**NIHSS**	8.3 ± 2.9	8.9 ± 3.8	8.6 ± 2.3	7.6 ± 2.2	0.567
**Nasogastric tube**	7 (20.0)	2 (15.4)	4 (40)	1 (8.3)	0.158
**ROAG**	13.5 ± 2.0	13.8 ± 2.0	13.4 ± 2.0	13.1 ± 2.0	0.688
**GUSS**	14.8 ± 2.8	14.8 ± 2.4	14.6 ± 3.5	15.1 ± 2.9	0.889

*Note*. Values are presented as number (%) or mean ± standard deviation

*Abbreviations*: SD = standard deviation; BMI = body mass index; COPD = chronic obstructive pulmonary disease; GCS = Glasgow Coma Scale; NIHSS = National Institutes of Health Stroke Scale; ROAG = Revised Oral Assessment Guide; GUSS = Gugging Swallowing Screen; WBC = white blood cell count

* = *p* < 0.05; ** = *p* < 0.01

ROAG scores ranged from 10 to 17 (mean 13.5 ± 2.0), with 57.1% (*n* = 20) indicating severe oral problems and 42.9% (*n* = 15) showing mild to moderate issues at baseline. Based on the GUSS, 37.1% (*n* = 13) had moderate to severe swallowing difficulty, while 62.9% (*n* = 22) had mild problems. At baseline, no statistically significant differences in ROAG or GUSS scores were observed between groups.

### Feasibility of oral care combined with NMES therapy

The O-NMES was feasible, as nearly all Group B participants (*n* = 9, 90%) completed all the intervention sessions without adverse events other than mild, transient skin redness in all 10 participants. The skin redness was mild and resolved within an hour after the intervention. The only participant who failed to complete all the interventions did so due to a COVID-19 infection and subsequent isolation, which is unrelated to O-NMES.

### Impacts of oral care and combined NMES intervention on swallowing function

**Fig. 4:**
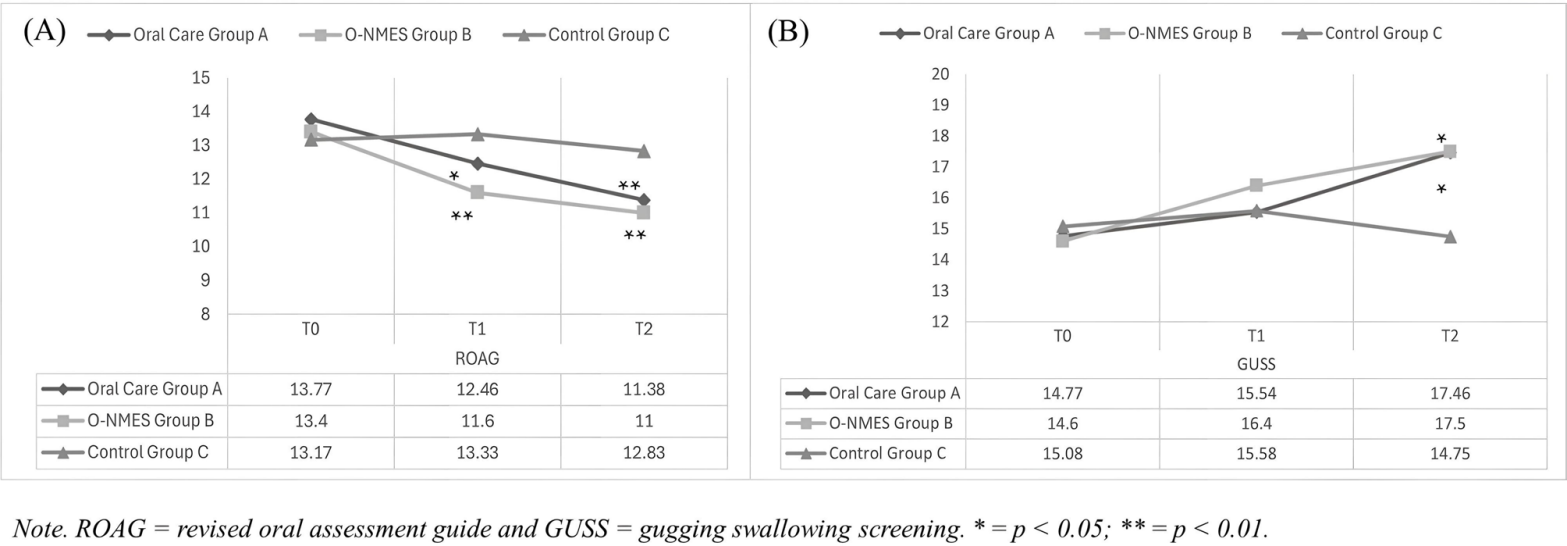
Score Changes Between Groups for (**A**) ROAG and (**B**) GUSS

The O-NMES intervention showed a positive impact on swallowing function. ROAG scores decreased at T1 and T2 in Groups A and B, while Group C showed a transient increase at T1, returning to baseline by T2 (Fig. 4-A). For Group A, scores decreased from 13.77 (SD = 2.05) to 12.46 (SD = 2.54) at T1, and 11.38 (SD = 2.69) at T2. Group B scores decreased from 13.40 (SD = 1.96) to 11.60 (SD = 2.07) at T1 and 11.00 (SD = 2.16) at T2. In contrast, Group C scores increased from 13.71 (SD = 2.04) to 13.33 (SD = 2.31) at T1 and returned to 12.83 (SD = 2.13) at T2 (Fig. 4-A). GEE analysis, adjusted for age and cardiovascular disease, showed statistically significant decreases in ROAG scores for Groups A and B at both T1 and T2 compared to Group C (Table 2).

**Table 2:** GEE results of intervention effects on ROAG and GUSS

	ROAG			GUSS		
	B	S.E.	P value	B	S.E.	P value
Intercept	8.608	1.9032	< 0.001	23.900	3.0539	< 0.001
O-NMES Group B ^a^	− 0.463	1.0973	0.673	2.589	1.7917	0.148
Oral care Group A ^a^	0.559	0.9469	0.555	1.280	1.5478	0.408
T2 ^b^	− 0.308	0.4866	0.527	− 0.092	0.8423	0.913
T1 ^c^	0.192	0.4866	0.693	0.742	0.8423	0.379
O-NMES Group B×T2 ^d^	-2.092	0.7160	0.003^**^	2.992	1.2404	0.016^*^
Oral care Group A×T2 ^d^	-2.077	0.6701	0.002^**^	2.784	1.1607	0.016^*^
O-NMES Group B×T1 ^e^	-1.992	0.7160	0.005^**^	1.058	1.2404	0.394
Oral care Group A×T1 ^e^	-1.500	0.6701	0.025^*^	0.028	1.1607	0.981
Coronary artery disease	0.305	0.9886	0.757	2.900	1.6352	076
Age	0.069	0.0299	0.021^*^	− 0.157	0.0481	0.001^**^

Note. *Abbreviations: GEE = Generalized Estimating Equations. ROAG = Revised Oral Assessment Guide; GUSS = Gugging Swallowing Screening*

a Reference value: Group C

b Reference value: T1

c Reference value: T0

d Reference value: Group C × T1

e Reference value: Group C × T0

All models were adjusted for age and cardiovascular disease. * *p* < 0.05; ** *p* < 0.01

### Impact of oral care and NMES intervention on the incidence of SAP

Among the 35 participants, four individuals (11.4%) developed SAP—all of whom were in the control group (Group C). No SAP cases were observed in either of the intervention groups (Groups A and B). Fisher’s exact test indicated a statistically significant difference in SAP incidence among the groups (*p* = 0.013; Table 3).

**Table 3:** Comparison of SAP incidence among groups

Group	Whole cohort (*n* = 35)	NMES with oral care (*n* = 10)	Oral care (*n* = 13)	Control (*n* = 12)	*p*-value
SAP, *n* (%)					
Yes	4 (11.4)	0 (0)	0 (0)	4 (33.3)	0.013*
No	31 (88.6)	10 (100)	13 (100)	8 (67.7)	

Note. *Abbreviations*: NMES = neuromuscular electrical stimulation; SAP = stroke-associated pneumonia. * *p* < 0.05, indicating significant differences among the three groups

## Discussion

This study is the first pilot RCT to assess the combined effects of NMES and oral care on swallowing function in hospitalized persons with AIS. It is also one of the few studies investigating early swallowing rehabilitation during the acute phase post-stroke. The study’s findings demonstrate the feasibility of the combined oral care and NMES intervention, with all participants completing the protocol without NMES-related adverse effects. The study findings further support the potential effectiveness of this combined intervention which is discussed in the following paragraphs.

### Demographic and baseline characteristics

Overall, the demographic profile closely resembled persons with AIS in Taiwan [39]. enhancing the external validity of the findings. The baseline characteristics were selected based on established risk factors for SAP. The assessment of these variables plays a crucial role in ensuring group comparability and controlling confounding factors that could influence outcomes. Our results indicate that group differences existed in terms of age and cardiovascular disease risk, while no group differences were observed in disease-related factors such as cognitive function, stroke severity, or feeding methods. Therefore, age and cardiovascular risk were controlled in the GEE analysis to minimize their impact on the outcomes.

Notably, although participants in the O-NMES group had the highest average age, they still demonstrated the greatest improvements in swallowing function. This suggests that the combined intervention may be particularly effective in older adults—who are at increased risk for SAP and frequently face barriers to timely access to multidisciplinary rehabilitation, especially in acute care settings [40, 41].

### Improvement of swallowing function

Both oral care and the combined intervention (O-NMES) group were associated with improved swallowing function and oral hygiene, as reflected in GUSS and ROAG scores. Although the combined intervention group (O-NMES) showed numerically greater changes, the study was not powered to detect statistically significant differences between intervention arms. These preliminary findings underscore the need for further hypothesis-driven trials in this area of research.

The observed effects—particularly in the O-NMES group—may be attributed to neurophysiological mechanisms specific to the acute post-stroke phase, a period marked by heightened neuroplasticity. NMES provides sensory input and motor stimulation that may promote cortical reorganization and enhance hyolaryngeal excursion, thereby improving airway protection and swallowing efficiency. Even in the absence of active swallowing exercises, NMES may facilitate neuromuscular activation and reduce disuse, especially when combined with sensory input from oral care [42–46].

Our findings are consistent with meta-analyses reporting the effectiveness of NMES in dysphagia rehabilitation. When implemented during the early post-stroke period, NMES may help prevent muscle atrophy and promote cortical engagement in persons with stroke who are not yet able to participate in active rehabilitation [47–49]. Oral care serves to reduce the oral bacterial load—a key modifiable risk factor for SAP [12, 50]. In line with previous findings, our results demonstrated that delivering both interventions within 48 h post-stroke is both feasible and clinically beneficial, particularly in settings with limited access to specialized rehabilitation services. Microbiological changes were not directly assessed in this study, and a structured oral care protocol provides a foundational step toward integrating oral hygiene into early dysphagia care.

These findings suggest that early-phase, nurse-led interventions may offer a pragmatic strategy to stimulate swallowing recovery, even passively. The improvements observed may reflect underlying neurophysiological adaptations, highlighting a window of opportunity that future studies should further explore.

### Prevention of SAP

In addition to functional improvements, both intervention have potential clinical implications in reducing the risk of SAP. While neither experimental group experienced SAP during the study period, compared to a 33.3% incidence in the control group, this pilot study was not powered to detect statistically significant differences between experimental groups. These findings suggest a possible preventive effect of oral care and NMES, either individually or in combination, on SAP development. To our knowledge, this is the first study to explore such an association in persons with acute stroke. Larger RCTs are needed to confirm these preliminary observations, and future research should evaluate cost-effectiveness and long-term quality-of-life outcomes to strengthen the evidence base for these interventions.

### Clinical implications and future directions

Early rehabilitation post-stroke poses challenges, often requiring intact cognitive function, swallowing ability, or specialized therapists. However, combining oral care and NMES offers a practical intervention deliverable within 48 h of stroke onset by trained nurses. Integrating these interventions into routine care is both feasible and beneficial. Although the Bass brushing technique is widely recommended for plaque removal in hospitalized older adults, up to 60% of elderly participants hospitalized for pneumonia are unable to perform it independently [51]. This highlights the critical need for nurse-led oral care protocols, particularly during the acute stroke phase when persons with stroke are most vulnerable and least able to perform self-care.

Although NMES is traditionally paired with active swallowing exercises, its proposed dual mechanism—providing both sensory input and motor stimulation—may facilitate cortical reorganization and reduce disuse atrophy, even in passive contexts [43, 52]. In the acute post-stroke phase, this mechanism could offer early neuromodulator input to support recovery when persons with stroke are unable to participate in active therapy.

Furthermore, the clinical applicability of this combined intervention may extend to persons with stroke who have more severe dysphagia, as evidenced by our findings. Nasogastric tube (NGT) placement, a marker of impaired swallowing and a known risk factor for aspiration pneumonia [53], was most prevalent in Group B. Despite this, participants in Group B demonstrated the greatest improvements in swallowing outcomes and experienced no SAP cases. These results support the potential of early NMES and oral care to mitigate complications even among individuals at high risk of aspiration.

While our study did not include persons with impaired consciousness, the noninvasive and protocolized nature of this intervention—delivered entirely by trained nurses without requiring active cooperation—suggests potential applicability to cognitively impaired or functionally limited populations. However, this application remains theoretical, and further research is needed to evaluate its feasibility and safety in these high-risk groups. Future studies should assess not only the clinical outcomes but also the real-world impact, cost-effectiveness, and scalability of this intervention in diverse care settings. Such investigations will help inform evidence-based guidelines and optimize early rehabilitation strategies for individuals recovering from acute ischemic stroke.

In conclusion, this pilot study underscores the promise of a nurse-led combining NMES and oral care intervention in acute stroke care. While the effectiveness of oral care is well established, the additional value of NMES warrants deeper investigation. Future large-scale, multicenter trials incorporating blinded assessments and objective swallowing evaluations are not just essential, but also an exciting opportunity to validate these findings and inform evidence-based stroke rehabilitation strategies.

### Limitations

This study has several limitations. First, the small sample size may affect statistical robustness and limit generalizability. Although adequate for detecting significant differences in primary outcomes, the study may have been underpowered to detect smaller effect sizes or subgroup variations. Future studies with larger sample sizes are needed to strengthen statistical power and enhance clinical applicability.

Second, due to human resource constraints in this pilot study, blinding of outcome assessors was not feasible, introducing a potential risk of assessment bias—particularly given the visible differences between intervention and control groups. Although standardized tools were used to reduce subjectivity, the lack of blinding may have influenced outcome ratings. Future studies should implement blinded protocols, including the use of sham NMES, to enhance internal validity and reduce assessment bias.

Third, the study was conducted at a single center, which may limit the generalizability of the findings to other institutions with different populations, staffing models, and clinical protocols. A multicenter study would help validate the intervention’s broader feasibility and effectiveness.

Additionally, participants eligible for intravenous rt-PA or mechanical thrombectomy were excluded because they required ICU-level care, where structured oral hygiene is routinely administered by nursing staff. This exclusion helped minimize intervention contamination and ensured consistency in usual care. However, it may limit the applicability of our findings to persons with more severe stroke who are admitted to the ICU.

There was also variability in nutritional intake methods—such as oral feeding versus NGT feeding—that could have introduced heterogeneity in clinical outcomes. Although GUSS-based dietary recommendations were provided (e.g., regular diet, soft diet, thickened fluids, or tube feeding), actual dietary textures were not consistently documented. Moreover, while all participants received nutritional consultations during hospitalization, standardized intake records were lacking, limiting our ability to evaluate the impact of diet on swallowing recovery and SAP risk. Future studies should better control and document feeding methods and dietary consistency.

Oral care was not standardized in the control group. Although nursing logs indicated an average brushing frequency of 0.63 times per day, variability among staff and lack of supervision may have introduced inconsistency in oral hygiene practices. Furthermore, oral and salivary microbiota—known contributors to aspiration pneumonia—were not assessed. Incorporating microbiological measures in future studies could provide a more comprehensive understanding of how oral care influences SAP risk.

Lastly, although GUSS offered a reliable bedside screening tool for swallowing function, instrumental assessments such as video fluoroscopic swallowing study (VFSS) or fiberoptic endoscopic evaluation of swallowing (FEES) were not included due to resource limitations. These tools are considered gold standards in dysphagia assessment and could provide detailed information on aspiration risk and swallowing physiology. Future studies should incorporate VFSS or FEES to enhance diagnostic precision and evaluate intervention efficacy more objectively.

In conclusion, while this pilot trial supports the feasibility and potential benefits of combining NMES and oral care in acute ischemic stroke rehabilitation, it also highlights key limitations. Addressing these limitations through larger, masked, multicenter trials with broader outcome measures will be essential to validate further and refine early dysphagia interventions in stroke care.

## Conclusion

This pilot study provides preliminary evidence that a combined oral care and NMES intervention is feasible, safe, and well-tolerated in the acute phase of ischemic stroke. Improvements in swallowing function and oral hygiene were observed in both intervention groups, and no cases of SAP occurred among participants who received the interventions during the study period. While these findings are encouraging, this pilot study was not powered to detect differences between intervention arms, and results should be interpreted with caution.

The integration of protocolized oral care and NMES—deliverable by nursing staff within 48 h of stroke onset—may offer a pragmatic strategy to support early dysphagia management, particularly in healthcare settings with limited access to specialized rehabilitation services.

Future large-scale, multicenter randomized trials incorporating blinded outcome assessments, instrumental swallowing evaluations (e.g., VFSS or FEES), and health economic analyses are warranted to further investigate these preliminary findings, explore subgroup responses, and assess cost-effectiveness in real-world stroke rehabilitation settings.

## Data Availability

No datasets were generated or analysed during the current study.

## Abbreviation

AIS: Acute Ischemic Stroke
ANOVA: Analysis of Variance
BUN: Blood Urea Nitrogen
CAD: Coronary Artery Disease
CDC: Centers for Disease Control and Prevention
COPD: Chronic Obstructive Pulmonary Disease
COVID-19: Coronavirus Disease 2019
CRP: C-Reactive Protein
FEES: Fiberoptic Endoscopic Evaluation of Swallowing
GCS: Glasgow Coma Scale
GEE: Generalized Estimating Equation
GUSS: Gugging Swallowing Screening
ICU: Intensive Care Unit
ITT: Intention-to-Treat
M: Means
NGT: Nasogastric Tube
NIHSS: National Institutes of Health Stroke Scale
NMES: Neuromuscular Electrical Stimulation
PP: Per-Protocol
PRS: Protocol Registration and Results System
RCT: Randomized Controlled Trial
ROAG: Revised Oral Assessment Guide
r-tPA: Recombinant Tissue Plasminogen Activator
SAP: Stroke-Associated Pneumonia
SD: Standard Deviations
VFSS: VideoFluoroscopic Swallowing Study
WBC: White Blood Cell
